# Multiple domains of functioning in older adults during the pandemic: design and basic characteristics of the Longitudinal Aging Study Amsterdam COVID-19 questionnaire

**DOI:** 10.1007/s40520-021-01829-8

**Published:** 2021-03-15

**Authors:** Emiel O. Hoogendijk, Marleen H. L. van der Horst, Jan Poppelaars, Majogé van Vliet, Martijn Huisman

**Affiliations:** 1 Department of Epidemiology and Data Science, Amsterdam Public Health Research Institute, Amsterdam UMC-Location VU University Medical Center, Amsterdam, The Netherlands; 2 Department of Sociology, Faculty of Social Sciences, Vrije Universiteit, Amsterdam, The Netherlands

**Keywords:** COVID-19, Cohort study, Epidemiology, Older adults, Coronavirus

## Abstract

The Longitudinal Aging Study Amsterdam (LASA) is an ongoing cohort study among older adults in the Netherlands. Respondents are usually interviewed approximately every 3 years. Because of the exceptional situation of the COVID-19 pandemic, it was decided to add an extra assessment in between, consisting of a postal/digital questionnaire with measures assessing the impact of the COVID-19 situation, as well as a selection of measures from regular LASA measurement cycles covering the physical, social and mental domains. In total, 1128 LASA respondents aged 62–102 years provided data, just after the first wave of the pandemic in 2020. This paper describes the methods and design of the LASA COVID-19 questionnaire, as well as the basic characteristics of the sample, including an overview of impactful situations experienced by older adults during the first months of the pandemic. The data of the questionnaire may be used to study the impact of the COVID-19 pandemic on multiple domains of functioning in older adults.

**Supplementary Information:**

The online version contains supplementary material available at 10.1007/s40520-021-01829-8.

## Introduction

In the first months of 2020, the coronavirus disease 2019 (COVID-19) caused by SARS-CoV-2 quickly spread across Europe. In the Netherlands, the first case of COVID-19 was diagnosed on February 27, 2020 [1]. In the following weeks, the number of new cases increased rapidly and the first COVID-19-related deaths were reported. In response to the quickly worsening public health situation, the Dutch government decided to implement a so-called “intelligent lockdown” [2]. As of March 12, citizens of the Netherlands were urged to stay at home and to only leave their homes when necessary (e.g., for grocery shopping). From March 15 onwards, restaurants, schools, gyms and close-contact professions, such as hairdressers, were closed. Older adults were advised to receive no visitors at home and it was no longer allowed to visit people in nursing homes. From mid-May, most measures were eased, primary schools re-opened as well as close-contact professions, and as of June 1 also restaurants re-opened. However, some measures remained in place, such as the advice to stay at home as much as possible, and to keep 1.5 m distance from other persons outside of the household.

Although individuals of all ages are at risk of COVID-19 [3], the consequences may be more severe in older adults, amongst others because of their higher rates of comorbidity [4–6]. It has also been suggested that older adults are disproportionally affected by lockdown policies, as these may increase loneliness, social isolation and the long-term risk of adverse health outcomes [7, 8]. To investigate the direct and indirect impact of the COVID-19 pandemic on health and functioning of older adults, it is important to follow them longitudinally, before, during and after the pandemic.

The Longitudinal Aging Study Amsterdam (LASA) is an ongoing cohort study among older adults in the Netherlands, initiated in 1992 [9–11]. The study started with a representative sample of older adults aged 55–84 years, which are interviewed approximately every 3 years. Exactly 10 and 20 years after baseline measurement, refresher cohorts aged 55–64 years were added to the study. LASA is a rich database on aging in the Netherlands, with information on multiple domains of functioning over an extended time period (see previous publications for more details [9–11]). However, with its 3-year follow-up schedule, the study is sometimes not able to fully capture the effects of impactful situations occurring between follow-up measurements. The current COVID-19 pandemic is an example of such situations. Just before the outbreak of the pandemic, we completed a measurement cycle (2018–2019). Since the COVID-19 pandemic is an exceptional situation, which may have serious consequences for the daily lives of older adults, we decided to add an extra assessment in between our 2018–2019 and our planned 2021–2022 measurement cycle. This assessment consisted of a postal questionnaire with measures that assess the impact of the COVID-19 situation, as well as a selection of measures from regular LASA measurement cycles covering the physical, social and mental domains of functioning.

The aim of the current paper was to describe the methods and design of the LASA COVID-19 questionnaire, as well as the basic characteristics of the sample, including impactful situations reported by Dutch older adults during the first months of the pandemic.

## Methods

### Sample and design

The LASA COVID-19 questionnaire was sent to LASA participants on June 8, 2020, just after the first COVID-19 wave in the Netherlands. Data were recorded between June 9, 2020 and October 8, 2020. However, 99% of all data were received before the end of August 2020. Of 1701 LASA respondents that were participating in the last measurement cycle (Wave J, 2018–2019), we selected 1485 respondents. Respondents that were not selected (*n* = 216), already had died (*n* = 61) or were purposely not selected (*n* = 155). The latter group includes people for whom the questionnaire was expected to be too much of a burden, such as for respondents who only did a short telephone interview or had a proxy interview at the last measurement cycle (Wave J). The 1485 respondents that were selected for the COVID-19 questionnaire received a questionnaire by postal mail. Respondents were also given the opportunity to fill out the questionnaire online (digital questionnaire). The oldest respondents (aged 80+) who did not respond and were not able to complete one of the other registration modes were offered to answer the questions in a telephone interview.

Of the 1485 LASA participants who received the questionnaire, 1128 (76%) participated. Registration modes were as follows: written questionnaire (*n* = 909), digital questionnaire (*n* = 198), and telephone interview (*n* = 21). Reasons for non-response were: no return of questionnaire/no answer (*n* = 250, 16.8%), refusal (*n* = 60, 4.0%), deceased before approach (*n* = 13, 0.9%), or ineligible (*n* = 34, 2.3%). Non-response analyses showed that people who did not participate in the LASA COVID-19 questionnaire were lower educated (education in years, mean = 10.8 versus 11.4, *p* < 0.01) and had lower cognitive scores (MMSE score, mean = 27.2 versus 28.2, *p* < 0.001) compared to those who participated. There were no statistically significant differences in age, sex, chronic diseases and functional limitations. The LASA study [9–11] received approval by the medical ethics committee of the VU University medical center. All participants provided written informed consent.

### Measures in the LASA COVID-19 questionnaire

An overview of the measures included in the LASA COVID-19 questionnaire is provided in Table 1. The questionnaire included measures specific for COVID-19, such as symptoms which were known to be associated with SARS-CoV-2 infection at the time, changes in access to healthcare, changes in social contact, and changes in diet and physical activity. The questions focus on the period since March 1, 2020. Most of these questions were newly developed, or came from existing population-based COVID-19 studies in the Netherlands [12]. Details on the COVID-19-related questions are provided in Online Supplementary data (Appendix 1). Additionally, the questionnaire included a selection of measures from regular LASA measurement cycles, covering multiple domains of functioning: self-rated health, functional limitations, social contact, loneliness, mastery, depression, anxiety, advanced care planning, provision of personal/household care and use of personal/household/nursing care [10, 11].

**Table 1:** Measures included in the LASA COVID-19 questionnaire

Measure	Instrument/details
1.COVID-19 related	
a. Symptoms	Questions on symptoms, diagnosis and hospitalization
b. Care wishes and quarantine	Questions on communication with healthcare professionals related to COVID-19 care, and quarantine details
c. Changes in healthcare use	Questions on the extent to which the COVID-19 situation has affected healthcare use
d. Changes in household/personal care	Questions on the extent to which the COVID-19 situation has affected provision and receiving of household/personal care
e. Changes in social contact	Changes in contact frequency with various types of personal relationships in the social network during COVID-19 pandemic
f. Nutrition and physical activity	Questions on changes in food consumption, alcohol use and physical activity
g. Work/employment	Questions on changes in employment and job characteristics during the COVID-19 pandemic
h. Impact of life events	Questions on the emotional experience of life events/situations during the COVID-19 pandemic
i. Changes in personal development and meaning in life	Questions on how the COVID-19 situation has changed perceptions related to meaning in life
j. Positive experience (qualitative data)	Open question on positive experiences during the COVID-19 pandemic
2. Self-rated health	General health, 1 item
3. Functional limitations	Disability in 7 common daily activities
4. Physical activity	Number of days per week with physical activity
5. Social contact	Contact frequency with various types of personal relationships in the social network
6. Loneliness	De Jong Gierveld loneliness scale
7. Mastery	Pearlin Mastery Scale
8. Depression	Center for Epidemiologic Studies Depression scale (CES-D) short version (10-item scale)
9. Anxiety	Hospital Anxiety Depression Scale-Anxiety subscale (HADS-A)
10. Advanced care planning	Questions on wishes regarding care at the end of life
11. Providing personal/household care	Questions on providing assistance with personal/household care
12. Use of personal/household/nursing care	Questions on receiving personal/household/nursing care

### Basic characteristics: measures

For the purpose of the current article, we provided an overview of the basic characteristics of the LASA COVID-19 questionnaire participants, as well as an overview of impactful life events during the first wave of the pandemic. Basic characteristics included age, sex, educational level, region and COVID-19 incidence among respondents and close relatives. The highest level of education attained was categorized as low (elementary school or less), medium (lower vocational or general intermediate education) or high (intermediate vocational education, general secondary school, higher vocational education, college or university). Region represents the three regions in which LASA respondents were recruited: the western part of the Netherlands (in and around Amsterdam, Water- and Wormerland), in the northeast (in and around Zwolle, Zwartewaterland and Ommen) and in the south (in and around Oss and Uden). The southern region was one the most affected areas in the Netherlands in the beginning of the pandemic, in terms of number of COVID-19 cases. COVID-19 incidence was based on self-report. Respondents were asked whether a doctor or other healthcare professional told them they probably had COVID-19, based on their symptoms, or whether they were tested positive for COVID-19. Respondents were also asked whether close relatives (partner, parent, child) were tested positive for COVID-19. We assessed the consequences of the COVID-19 pandemic and intelligent lockdown with the following question: “Are there any events in your life that occurred because of the COVID-19 crisis that strongly affect you?” This was followed by 10 different situations or life events, such as experiencing illness, having no/less contact with children or grandchildren, or experiencing financial difficulties. Response categories were: (1) strong impact, (2) moderate impact or (3) no impact.

### Statistical analysis

Descriptive analyses were done in SPSS 26 (IBM Corp, Armonk, NY, USA). Mean (SD) and proportions were reported for basis characteristics and impactful situations.

## Results

The basis characteristics of the LASA COVID-19 sample are shown in Table 2. Participants were on average 73.9 years old, the majority were female (52.8%) and higher educated (54.6%). In this sample, 29 respondents (2.6%) reported a COVID-19 infection, of which only one (0.2%) was based on a positive test, and 40 respondents (3.5%) had a close relative with a positive test result.

**Table 2:** Characteristics of the study sample (*n* = 1128)

Characteristics	Mean (SD) or *n* (%)
Age, 62–102 years, mean (SD)	73.9 (7.5)
<75 years, *n* (%)	689 (61.1)
≥75 years, *n* (%)	439 (38.9)
Sex (female), *n* (%)	596 (52.8)
Educational level	
Low, *n* (%)	132 (11.7)
Medium, *n* (%)	380 (33.7)
High, *n* (%)	616 (54.6)
Region	
Amsterdam, *n* (%)	497 (44.1)
Zwolle, *n* (%)	371 (32.9)
Oss, *n* (%)	260 (23.0)
COVID-19 infection	
Yes (told by healthcare professional or based on diagnostic test), *n* (%)	29 (2.6)
COVID-19 infection close relative (partner, parent, child)	
Positive diagnostic test, *n* (%)	40 (3.5)

**Fig. 1:**
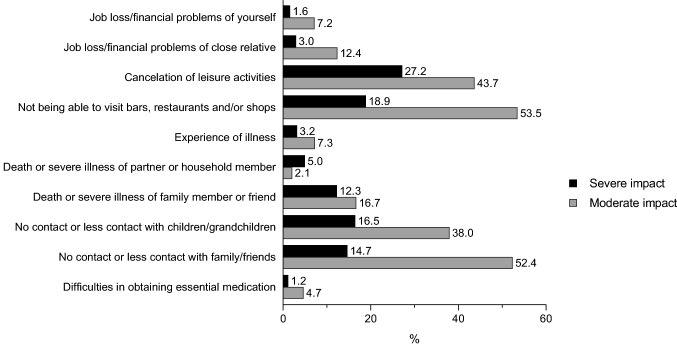
Impact of situations and life events during the first wave of the COVID-19 pandemic, as reported by Dutch older adults

Figure 1 provides an overview of impactful situations that were assessed. The most frequently identified situations during the first months of the pandemic with strong or moderate impact on older adults were: cancelation of leisure activities (strong: 27.2%, moderate: 43.7%), not being able to visit bars, restaurants and/or shops (strong: 18.9%, moderate: 53.5%), no contact or less contact with children/grandchildren (strong: 16.5%, moderate: 38.0%), no contact or less contact with family/friends (strong: 14.7%, moderate: 52.4%), and death or severe illness of family member or friend (strong: 12.3%, moderate: 16.7%).

## Discussion

In this paper, we provided an overview of the design and measures of the LASA COVID-19 questionnaire, an extra measurement of the LASA study that aims to capture the impact of the COVID-19 pandemic on various domains of functioning in Dutch older adults. Furthermore, we presented the basic characteristics of the study sample, including impactful situations during the first months of the pandemic. The results showed that participants reported moderate to strong impact of the COVID-19 situation on their ability to perform leisure activities, not being able to visit bars, restaurants and shops, decrease in contact with family or friends, and for death or illness of close relatives.

In the current study, the incidence of COVID-19 reported by older adults during the first months of the pandemic was 2.6%. Based on a positive test, the incidence was even lower (0.2%), even though a substantial number of our respondents live in areas in the Netherlands that were relatively strongly affected at the time by COVID-19. Other population-based studies, such as the UK Biobank, also reported low numbers of positive cases in the first month of the pandemic (0.2%), mainly because people were only tested in case of severe symptoms [5].

The data of the LASA COVID-19 questionnaire provide great opportunities to study the impact of the COVID-19 pandemic on multiple domains of functioning in older adults. This includes cross-sectional studies in which various COVID-19-specific measures can be related to physical, social and mental functioning, but also longitudinal studies. The data of the COVID-19 questionnaire may be linked to historical data of LASA, that covers a period of 28 years of longitudinal follow-up on a broad range of measures [10, 11], and to data that will become available in future LASA measurement cycles. For example, historical trajectories of depression or frailty [13, 14] may be linked to outcomes measured during the COVID-19 pandemic.

The LASA COVID-19 questionnaire has some limitations that should be acknowledged. First, when longitudinal analyses are done that includes data of this questionnaire, researchers should be aware of a potential mode effect. Compared to previous LASA measurement cycles, the administration mode of the measures differs sometimes. Usually, data are collected in face-to-face interviews including clinical tests, and only a part is collected in a written questionnaire. For the COVID-19 questionnaire, all measures are self-reported and mostly based on a written questionnaire. Second, we only have data available from participants that were healthy enough to fill out the questionnaire. Therefore, some groups may have been underrepresented in the sample, such as the oldest old. If respondents were severely ill or even died because of COVID-19, we do not always have this information. Links with mortality registration data (including cause of death) have not yet been established for those who recently died, but will become available in the future. Third, the questionnaire includes a selection of measures from regular LASA measurement cycles and from multiple domains of functioning, but some measures (e.g., CES-D) were shortened. Finally, some new measures that were included to specifically study the impact of the COVID-19 situation may not be studied longitudinally. However, they could be related to various long-term outcomes with data from future LASA measurement cycles.

In conclusion, the data of the LASA COVID-19 questionnaire provide a unique opportunity to study the impact of the COVID-19 pandemic on multiple domains of functioning in community-dwelling older adults, cross-sectionally and longitudinally. This includes COVID-19-specific measures, as well as assessments of physical, social and mental functioning. In this paper, we provided some initial results. More findings will become available in the near future, as the data are currently being analyzed in various research projects. The data are also available for analysis by external researchers and can be requested (see www.lasa-vu.nl for data request procedures).

## Supplementary Information

Below is the link to the electronic supplementary material.

Supplementary file1 (PDF 119 KB)

## Data Availability

The datasets generated during and/or analyzed during the current study are not publicly available due to confidentiality, but the data underlying the results presented in this study are available from the Longitudinal Aging Study Amsterdam (LASA). Data of LASA, including data from the LASA COVID-19 questionnaire, may be requested for research purposes. More information on data requests can be found on the LASA website: www.lasa-vu.nl.
