# Supplementary material for: Multiple domains of functioning in older adults during the pandemic: design and basic characteristics of the Longitudinal Aging Study Amsterdam COVID-19 questionnaire
Source: Aging Clin Exp Res. 2021 Mar 15;33(5):1423–8. doi: 10.1007/s40520-021-01829-8 (PMC7957282; doi:10.1007/s40520-021-01829-8)
Supplement: Supplementary file 1 — Supplementary file1 (PDF 119 KB) [file 40520_2021_1829_MOESM1_ESM.pdf]

APPENDIX 1. COVID-19 related questions included in the LASA COVID-19 questionnaire

Covid-19 symptoms, diagnosis and hospitalization

Various questions were asked on symptoms, diagnosis and hospitalization.

- Respondents were asked whether they had (since March 1, 2020) one or more of the following symptoms: fever, fatigue, dry cough, shortness of breath, coughing up sticky mucus, joint pain, sore throat, headache, cold shivers, nausea or vomiting, stuffy or running nose, loss of smell, loss of taste, diarrhea, coughing up blood, and passing out. Answering categories for each symptom: (1) No, (2) A little, (3) Moderate, (4) Severe, and (5) Don`t know.
- Did a doctor or other healthcare professional tell you that you probably had COVID-19, based on your symptoms? Answering categories: (1) Yes, and (2) No.
- Have you been tested for covid-19? Answering categories: (1) Yes, and (2) No. If yes, the respondent was asked for the date and test result.
- Do you think you should have been tested for covid-19? Answering categories: (1) Yes, (2) No, and (3) Don`t know.
- Have you been hospitalized because of (possible) covid-19? Answering categories: (1) Yes, and (2) No.
- Have you been admitted to an intensive care unit because of covid-19? Answering categories: (1) Yes, and (2) No.
- Do you know anyone who tested positive for covid-19, was hospitalized for covid-19 or died because of covid-19 since March 1, 2020? For each of the following types of personal relationships this was asked: partner, parent, child, sibling, grandchild, other family member, neighbor, friend/acquaintance, and other. Positive test, hospitalization and death were administered separately.

Care wishes and quarantine

Various questions were asked on communication with healthcare professionals related to covid-19 care, and quarantine details.

- As of March 1, 2020, did you talk with a healthcare professional about what treatment options are available for you in case of severe illness? Answering categories: (1) Admission to intensive care unit, (2) Mechanical ventilation, (3) Reanimation, (4) None of these.
- Did you talk with a healthcare professional about whether admission to an intensive care unit would be appropriate or desirable for you? Answering categories: (1) Yes, and (2) No.
- Did a doctor or healthcare professional tell you that you should self-quarantine? Answering categories: (1) Yes, and (2) No. If yes, for how many days have you been in quarantine?
- Did you decide yourself to self-quarantine? Answering categories: (1) Yes, and (2) No. If yes, for how many days have you been in quarantine?

Changes in healthcare use

Various questions were asked on delay, postponement, adaptation, and avoidance of (medical) care.

Did you, because of the covid-19 situation, ….

- Experience a GP-initiated cancelation of an appointment?
- Experience a hospital-initiated cancelation of a scheduled appointment with a medical specialist?
- Have a telephone consultation with the GP instead of a visit to the GP?
- Have a telephone consultation with a medical specialist instead of a visit to the outpatient clinic?
- Cancel a scheduled visit to the GP yourself?
- Cancel a scheduled outpatient appointment with a medical specialist yourself?

Answering categories: (1) No, (2) Yes, and (3) Not applicable, I did not have an appointment.

Did you, because of the covid-19 situation, …..

- Decide not to contact the GP, even though you would like to?
- Decide not to contact a medical specialist, even though you would like to?
- Postpone help-seeking in case of physical or mental health complaints?
- Call a telephone support line, such as an older persons support line or the Red Cross support line?

- Contact your GP practice with questions regarding covid-19?

Answering categories: (1) No, (2) Yes, and (3) Don`t know / not applicable.

Changes in household/personal care

Several questions were asked on the extent to which the covid-19 situation has affected provision and receiving of household/personal care.

- For those who provide care to others: Overall, did the care burden increase, decrease or remain the same during the covid-19 situation? Answering categories: (1) Much less, (2) Less, (3) Not more, not less, (4) More, (5) Much more, and (6) Not applicable.
- For those who provide care to others: Overall, because of the covid-19 situation, do you provide less or more household care / personal care? Answering categories: (1) Much less, (2) Less, (3) The same as before, (4) More, (5) Much more, and (6) Not applicable.
- Did you receive less household/personal/nursing care since March 1, 2020, because of the covid-19 situation? Answering categories: (1) Not applicable, I don`t receive any care, (2) No, I receive the same amount of care, and (3) Yes, I receive less care than before March 1, 2020.
- If yes, please indicate from whom you receive less care? Answering categories: (1) Less care from partner, family, neighbors or friends, (2) Less care from volunteers, (3) Less care from professional caregivers (community nurse, homecare, institutional care), and (4) Less care from paid home help.

Changes in social contact

Respondents were asked about contact with people outside of their household. With contact we mean visits (people who visit you, or you visiting other people), telephone contact, email contact, WhatsApp contact, or video-calling.

1. How often did you have contact with these persons in the past few weeks?
2. Is the contact now, during the covid-19 pandemic, different than before?

These two questions were answered for each type of personal relationship: children (outside of your household), grandchildren (outside of your household), sons-in-law/daughters-in-law, other family

members, friends and acquaintances, neighbors, shop assistants, community nurse/home care staff, cleaner, and pastor/spiritual counsellor.

Nutrition and physical activity

Respondents were asked about perceived changes in nutrition behaviors and physical activity behaviors in the past few weeks.

Did you, because of the covid-19 situation, ….

- Experience difficulty obtaining groceries?
- Skip warm meals?
- Eat less than normal?
- Have less physical activity than normal?
- Exercise less than normal?

To what extent do you, because of the covid-19 situation, ….

- Have the feeling that you eat too little or lose weight?
- Have the feeling that you don`t have enough physical activity or exercise?
- Have the feeling that you gain weight?
- Have the feeling that you snack more than you normally do?
- Have the feeling that you drink more alcoholic beverages than you normally do?

Answering categories: (1) Always, (2) Sometimes, (3) Never, (4) Don`t know

Work / employment

Respondents were asked whether they had paid employment before March 1, 2020. Furthermore, they were asked how many hours they worked per week and what type of contract they had. Next, they were asked about whether anything has changed in their work situation since the implementation of the government measures to control the covid-19 situation. If yes, they were asked about details regarding their current employment status, number of working hours per week and type of contract. Finally, a question was asked about whether changes in employment had an effect on the received wages. Answering categories were: (1) Salary is the same, because of compensation (by employer or

government), (2) Salary is lower, because I work less hours, (3) Salary is lower, because of salary reduction, (4) Other: …. , and (5) Not applicable.

Impact of life events

Respondents were asked about the impact of situations and life events related to the COVID-19 situation or lockdown policies.

Are there any situations or events in your life that occurred because of the COVID-19 crisis that strongly affect you?

- Job loss/financial problems of yourself
- Job loss/financial problems of close relative
- Cancelation of usual leisure activities, such as participating in organizations or associations
- Not being able to visit bars, restaurants and/or shops
- Experience of illness (own illness)
- Death or severe illness of partner or household member
- Death or severe illness of family member or friend
- No contact or less contact with children/grandchildren
- No contact or less contact with family/friends
- Difficulties in obtaining essential medication

Answering categories: (1) Strong impact, (2) Moderate impact, and (3) No impact.

Changes in personal development and meaning in life

Respondents were asked about changes in situations related to personal development and meaning in life.

During the covid-19 pandemic, daily life has changed substantially. Please indicate for the following items what is applicable to your situation?

- Paying attention to the things I enjoy doing in my spare time
- Paying attention to my religion
- Making plans for the future

- Paying attention to my personal development
- Reflecting on what is really important in my life

Answering categories: (1) Much more than before the outbreak of covid-19, (2) More than before the outbreak of covid-19, (3) Not more and not less than before the outbreak of covid-19, (4) Less than before the outbreak of covid-19, and (5) Much less than before the outbreak of covid-19.

Positive experience (qualitative data)

Open question: Can you mention at least one positive aspect of your experience of the covid-19 situation? If yes, please describe this?

ADDITIONAL INFORMATION

Please note that the LASA COVID-19 questionnaire is administered in Dutch. The questions above have been translated from Dutch to English by the researchers. This is just for illustrative purposes. We did not display measures from regular LASA measurement waves, as details of these measures can be found on the study website: www.lasa-vu.nl
